# Case report: Experience and insights on the treatment of two cases of cryptococcal meningitis during the later stages of the COVID-19 pandemic

**DOI:** 10.3389/fimmu.2024.1361277

**Published:** 2024-04-22

**Authors:** Yuli Zhou, Bingfeng Qiu, Jun Jiang, Tianwen Chen, Liqian Wang, Yunxing Yang, Senlin Ruan, Yanlei Chen, Huanli Fang, Juan Jin, Nan Yang

**Affiliations:** ^1^ Department of Laboratory Medicine, Affiliated Hangzhou First People’s Hospital, Westlake University School of Medicine, Hangzhou, China; ^2^ Department of Laboratory Medicine, People’s Hospital of Tonglu County, Hangzhou, Zhejiang, China; ^3^ Marketing Department, Guilin URIT Medical Electronic Co., Ltd., Guilin, Guangxi, China; ^4^ Department of Neurology, Affiliated Hangzhou First People’s Hospital, Westlake University School of Medicine, Hangzhou, China; ^5^ Department of Nephrology, the First Affiliated Hospital of Zhejiang Chinese Medical University (Zhejiang Provincial Hospital of Traditional Chinese Medicine), Hangzhou, Zhejiang, China

**Keywords:** novel coronavirus, cryptococcal meningitis, opportunistic infections, cerebrospinal fluid morphology, laboratory examinations

## Abstract

In the late stages of the COVID-19 pandemic, there’s an increasing trend in opportunistic infections, including bacterial and fungal infections. This study discusses the treatment process of two cases of cryptococcal meningitis during the COVID-19 pandemic. It highlights the importance of laboratory testing for these co-infections and stresses the need for vigilance, early diagnosis, and proactive treatment to improve patient outcomes in the post-pandemic era.

## Introduction

Since the outbreak of the novel coronavirus in 2019, COVID-19 continues to be a focal issue in the global public health arena (1).This virus is a ribonucleic acid (RNA) virus capable of causing severe infections accompanied by various complications. Under the influence of COVID-19, older individuals and those with compromised immune systems are more susceptible to opportunistic fungal infections. The immunosuppressive effects of COVID-19, coupled with the use of immunosuppressive medications in critically ill patients, have continually elevated the risk of opportunistic infections. These pathogens exacerbate the severity of the disease and increase the infection and mortality rates among global COVID-19 patients (2). There have been reported cases indicating the occurrence of cryptococcosis in patients infected with SARS-CoV-2, manifesting as cryptococcal meningitis(CM)or pneumonia. Currently reported cases of cryptococcosis in COVID-19 patients have been observed not only in individuals with compromised immune function but also in those with normal immune function (3, 4).

Cryptococcal infection is an opportunistic infection caused by *Cryptococcus neoformans* or *Cryptococcus gattii (* 5). *Cryptococcus* reproduces asexually as sterile filamentous single-budded yeasts, lacking capsules or having only small ones in the environment. However, upon entering the human body, they rapidly develop thick capsules. The presence of capsules significantly increases the diameter of *Cryptococcus* spores and enhances their pathogenicity. Initial infection of *Cryptococcus neoformans* occurs via inhalation of infectious propagules and subsequent colonization of the respiratory tract (6).When the immune system is compromised, the primary infection occurs in the lungs, then it disseminates and invades the central nervous system, resulting in CM as the most significant and commonly affected location (7, 8). Furthermore, there are pulmonary cryptococcosis. they have the capability to invade bones, muscles, and mucous membranes of the skin, giving rise to chronic inflammation and abscess formation. *Cryptococcus* is widely distributed in the natural environment, and susceptible individuals include those with chronic illnesses or patients on long-term immunosuppressive, cytotoxic, or glucocorticoid therapy.

In this case report, we present the treatment of two patients with SARS-CoV-2 infection complicated by *Cryptococcus neoformans* during the post-pandemic period. This underscores the critical role of laboratory testing in accurately diagnosing whether an individual has COVID-19, *Cryptococcus* infection, or a co-infection of both. In COVID-19 patients, it is crucial to enhance pathogen detection to prevent concurrent infections with rare pathogens. Timely diagnosis and treatment of CM patients, along with preventive measures against COVID-19, contribute to improving patient outcomes.

This study focuses on determining cases of *Cryptococcus* infection in the post-pandemic period and explores the observation and treatment experiences of CM cases occurring in the later stages of the COVID-19 pandemic. Case 1 has no history of COVID-19 infection or vaccination, which is crucial for understanding the susceptibility of *Cryptococcus* in the absence of exposure to the coronavirus. On the other hand, Case 2 not only has a history of COVID-19 infection but also underlying conditions like hypertension and diabetes, emphasizing the importance of these factors in understanding the dynamics of *Cryptococcus* infection post-pandemic. We emphasize that studying these two cases aligns with the background and theme of the research, as they provide unique experiences and treatment insights into *Cryptococcus* infection during the later stages of the COVID-19 pandemic, offering valuable insights into the potential relationship between COVID-19 and subsequent *Cryptococcus* susceptibility.

## Case presentation

### Case 1

**Figure 1:**
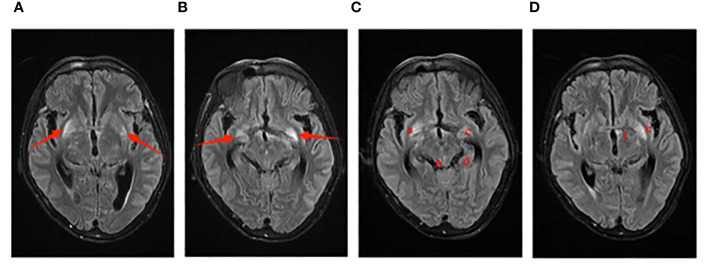
Imaging manifestations of Case 1 on cranial MRI. The T2 Flair sequence is as follows: Images **(A, B)** display small patchy abnormal signal foci symmetrically located at the base of the bilateral basal ganglia on January 4th. Images **(C, D)** reveal multiple abnormal signal shadows on the cranial MRI on January 10th, including the bottom of the bilateral basal ganglia (a, c), the globus pallidus (e), the cingulate gyrus of the temporal lobe (d), the left edge of the third ventricle (f), and the cerebral aqueduct (b).

A 47-year-old male was admitted with a history of palpitations and fatigue persisting for 10 years and a recent headache lasting 10 days. He has a medical history of hypertension, diabetes, hyperthyroidism, and heart disease. He has no family history of hereditary diseases, smoking, or alcohol consumption. He is allergic to strawberries. The patient has no history of previous COVID-19 infection, denies having visited any COVID-19 outbreak areas or countries in the past 14 days, and has had no direct or indirect contact with individuals from those regions. There has been no recent travel abroad, no involvement in any clustered outbreak events, and no contact with individuals exhibiting fever symptoms. Additionally, the patient has not received the COVID-19 vaccine. Physical examination revealed thyroid enlargement, irregular heart rhythm, and bilateral lower limb edema. Laboratory tests indicated a high white blood cell(WBC)count, abnormal neutrophil percentage, elevated high-sensitivity C-reactive protein, and positive results for syphilis and cryptococcal infections. The head magnetic resonance imaging (MRI) revealed a potential brain-related condition as shown in **Figure 1**. Analysis of the cerebrospinal fluid (CSF) indicated abnormalities, including a positive finding for cryptococcal infection. Utilizing CSF microscopy examination, morphological analysis can be conducted to diagnose *Cryptococcus* infection using India ink staining and Wright-Giemsa stain. Confirmation of the *Cryptococcus* species could be obtained through microbial culture and mass spectrometry identification. The final results showed a medium quantity of *Cryptococcus neoformans* in the CSF. Based on the comprehensive examination results, the diagnosis of cryptococcal intracranial infection has been confirmed. Initially, treatment was initiated with amphotericin B cholesterol sulfate complex and fluconazole. On the third day, the patient developed symptoms consistent withCOVID-19 and tested positive for SARS-CoV-2 using nucleic acid testing (PCR). The condition rapidly deteriorated, leading to multiple systemic complications. The medical team discontinued amphotericin B cholesterol sulfate complex and fluconazole, switching to voriconazole, and initiated antiviral therapy with remdesivir. Despite aggressive antiviral and resuscitative treatments, the patient unfortunately passed away on the 13th day of admission due to respiratory failure.

### Case 2

**Figure 2:**
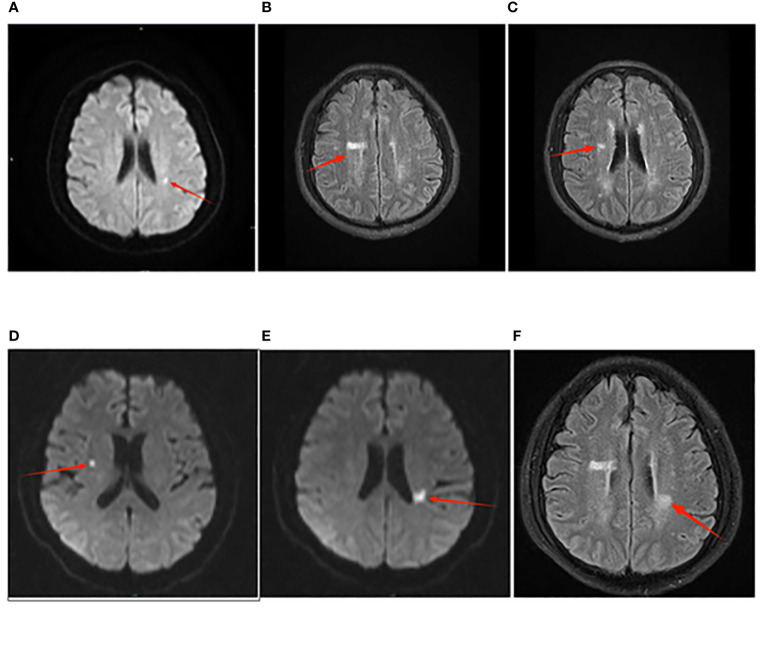
The presentation of cerebral infarction in Case 2 on the MRI images. Panel **(A–C)** represents the examination results from April 30th. In Panel **(A)**, the diffusion-weighted imaging (DWI) sequence shows punctate high signal foci adjacent to the left lateral ventricle, indicating acute cerebral infarction. In Panel **(B)**, T2 Flair sequence reveals patchy high signals adjacent to the right lateral ventricle. In Panel **(C)**, T2 Flair sequence shows patchy high signals adjacent to the right lateral ventricle. Panel **(D–F)** represents the examination results from May 23rd. In Panel **(D)**, T2 Flair sequence reveals punctate high signals adjacent to the right lateral ventricle. In Panel **(E)**, the T2 Flair sequence demonstrates patchy high signal intensities adjacent to the left lateral ventricle, particularly in the posterior horn region. In Panel **(F)**, there are also patchy high signal areas observed near the left lateral ventricle on the T2 Flair sequence.

**Figure 3:**
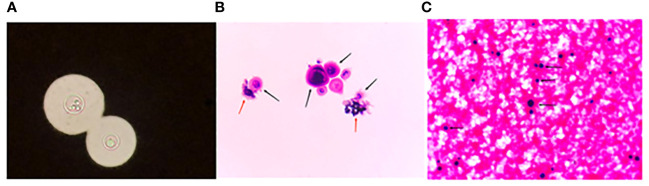
**(A)** displays CSF stained with India ink, magnified 1000x. Due to the lack of staining on the capsule, *Cryptococcus* appears as a “starry sky” against the ink background, with transparent spore bodies and prominent, thick capsules. **(B)** displays the CSF stained with Wright-Giemsa staining at a magnification of 1000x. *Cryptococcus* spores display an irregular size and may present as either circular or “8”-shaped structures, showing staining in various hues of purple-black or mauve. Spore bodies show purple-black or mauve pigmentation, while their enclosing capsules are either lightly pigmented or colorless. Prior to treatment, the spores exhibit vigorous growth, with radial fluff covering their surface, as indicated by the black arrows. The dense fluff is prone to contamination by residual dyes, which are difficult to remove, as indicated by the red arrow. **(C)** displays a Gram-stained smear of a positive blood culture from one side, magnified 400x. In the image, fungal spores are clearly visible, as indicated by the black arrows. These spores have a smooth surface and lack capsules. India ink staining yielded a negative result, but identification following inoculation on blood agar confirmed the presence of *Cryptococcus neoformans*.

A 52-year-old male, previously infected with COVID-19, has a history of hypertension and pancreatitis. He has a smoking and alcohol consumption habit, with no family history of hereditary diseases, and no history of drug or food allergies. He has received the COVID-19 vaccine before. He was admitted due to a four-day history of headache and two days of dizziness. Prior to admission, he had received ceftriaxone for infection control, amlodipine for blood pressure management, and mannitol for intracranial pressure reduction, but his condition did not improve. Physical examination revealed increased neck stiffness and a positive Kernig sign on both sides. Laboratory tests indicated elevated WBC count, increased neutrophil percentage, accelerated erythrocyte sedimentation rate, with all other blood parameters falling within the normal range. Chest CT revealed bronchitis, diffuse fibrous proliferation in both lungs, and mild thickening of the bilateral pleura. The head MRI revealed an acute cerebral infarction adjacent to the left lateral ventricle, as shown in **Figure 2**. accompanied by increased intracranial pressure of 400+ mmHg (normal range: 80-180 mmHg). The cerebrospinal fluid (CSF) routine test showed that the appearance was colorless and transparent, with a nucleated cell count of 31/μL. The Pandy test was weakly positive, and the CSF India ink staining indicated the presence of *Cryptococcus.* Further culture and identification of bacteria and fungi in the CSF revealed the detection of *Cryptococcus neoformans*. *Cryptococcus neoformans* was detected in blood culture, as depicted in **Figure 3**. Peripheral blood cultures from both hands were cultured for aerobic and anaerobic bacteria. After incubation, no bacterial growth was observed in the anaerobic bottles and one of the aerobic bottles; however, positive growth triggering an alarm was noted in the other aerobic bottle. Gram staining revealed fungal spores, as shown in **Figure 3C**. Subsequent subculturing on specific agar mediums confirmed fungal growth and identified a *Cryptococcus neoformans* strain through mass spectrometry. The patient had a *Cryptococcus neoformans* infection, ultimately diagnosed as CM. A four-week course of antifungal therapy is being conducted, which includes amphotericin B with flucytosine. In addition, adjunctive therapies such as mannitol, glycerol fructose, and albumin dehydration are being used to reduce intracranial pressure, alleviate symptoms, and prevent certain complications like brain herniation.

**Figure 4:**
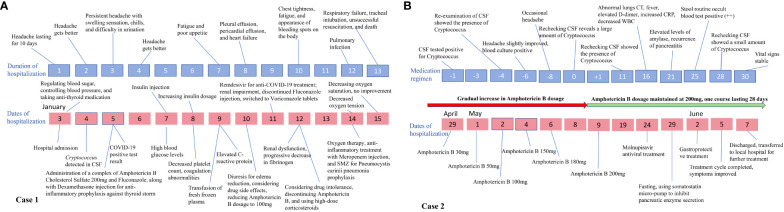
Timeline of changes in the condition of the two cases. **(A)** provides an illustration of how the symptoms and overall disease condition of case 1 evolved over time with increasing duration of hospital stay, along with depicting the implemented therapeutic interventions during this period; **(B)** shows the application of treatment plans and changes in disease condition over time for case 2 during hospitalization.

On the 16th day of antifungal treatment for Cryptococcal infection, the patient developed fever and symptoms of a pulmonary infection. Upon examination, signs of a lung infection were detected. The patient was initially infected with the novel coronavirus approximately 5 months ago, and they are currently experiencing a surge in secondary COVID-19 infections. Due to the patient’s compromised immune system and the potential for secondary infections, the doctor has opted to incorporate a 5-day course of Molnupiravir as part of the antiviral treatment plan. On day 21 of the treatment, the patient developed acute pancreatitis, confirmed by abdominal CT scans. Treatment includes fasting, omeprazole, and somatostatin to control pancreatic enzyme secretion. After completing a 4-week course of treatment for CM, the patient’s symptoms showed significant improvement. He has been discharged and will continue treatment at a local hospital. The treatment timelines for Case 1 and Case 2 are shown in **Figure 4**.

## Discussion

Patient 1 had various risk factors for *Cryptococcus* infection, such as prior illnesses, poor health, and weakened immunity. Their 10-day delay in seeking medical care worsened the situation. After admission, the patient was diagnosed with CM through various tests and received treatment. However, three days after admission, the patient developed chills and a fever, initially thought to be medication side effects. Later, a positive COVID-19 test was confirmed, and the patient experienced urinary issues, ongoing headaches, and altered consciousness. Additionally, there was a continuous drop in platelet count and the development of a hypercoagulable state, complicating diagnosis and treatment. The patient’s condition worsened rapidly, leading to multi-organ failure and eventual death. This complex case involved medication side effects resembling COVID-19 symptoms, and the interaction between COVID-19 and the cryptococcal infection led to severe complications.

In Case 2, the patient had a history of hypertension, pancreatitis, and a prior COVID-19 infection five months ago. They sought prompt medical care when symptoms appeared and were admitted after receiving treatment elsewhere. Tests confirmed a *Cryptococcus neoformans* infection affecting the blood and central nervous system. Considering the positive result of blood culture on one side, lack of fever and symptoms of multi-organ involvement, a transient bloodstream infection is initially suspected. However, on the 16th day of treatment, the patient developed fever, decreased white blood cell count, and lung inflammation. Considering the patient’s medical history and environmental factors, we have now taken into account the potential impact of COVID-19 and opted to commence preventive treatment for the virus. Following the administration of preventive anti-COVID-19 treatment, there has been improvement in the patient’s lung infection, confirming our initial suspicion. On the 21st day, pancreatitis recurred but was promptly managed. The treatment lasted 28 days, with significant symptom improvement, and the patient was successfully discharged.

Both Case 1 and Case 2 had CM, but Case 1’s treatment failed while Case 2 succeeded due to the following reasons: ①Health Differences: Case 1 had multiple health issues, poor health, and a weakened immune system, making them more vulnerable. In contrast, Case 2 was in better health. ②Timing: Case 1 delayed seeking help by 10 days, contributing to treatment failure. Case 2 sought timely treatment, effectively managing the condition. ③Immunity and the Role of Vaccines: Patient 1 was a first-time infection of the COVID-19, with no prior vaccination. After contracting COVID-19 in addition to CM, severe complications arose, and the condition rapidly deteriorated. On the other hand, Patient 2 experienced a secondary infection but had previously been vaccinated following the prevention and control policies. This vaccination helped alleviate the symptoms of the COVID-19 infection to some extent. Moreover, proactive treatment measures were taken, successfully preventing the occurrence of complications. ④Treatment Challenges: Patient 1 contracted COVID-19 during hospitalization, and the side effects of the medications were similar to the symptoms of the infection, complicating the symptoms and increasing the difficulty of treatment. On the other hand, Patient 2 received preventive treatment for COVID-19, effectively controlling the condition in a timely manner.

CM is an opportunistic infection with a high mortality rate (9). In the post-COVID-19 era, heightened attention to this disease is imperative. Currently, there is limited research on the co-infection of SARS-CoV-2 and CM (10). There have been numerous case reports and small reviews describing cryptococcal infection following COVID-19 (11, 12). The virus-induced excessive inflammatory immune response typically plays a central role in the pathogenesis of CM. Following SARS-CoV-2 infection, patients with cryptococcosis exhibited a mortality rate of 59%, which surpasses that of contemporary Cryptococcus cohorts. An evident correlation was observed between compromised immune status and the presentation of cryptococcal disease, along with mortality (13). Furthermore, the study emphasizes the necessity of conducting clinical and laboratory assessments for opportunistic infections for more than 30 days when relevant symptoms develop. *Cryptococcus* infection was predominantly observed in individuals with severe cases of COVID-19 who were treated with corticosteroids and hospitalized in the intensive care unit (12). There have been reports suggesting a potential association between the administration of steroids and tocilizumab in patients with COVID-19 in 2019, which may potentially contribute to the onset of CM (14–16).Research indicates that reduced T-cell response may drive CM. Given potential effects of SARS-CoV-2 on T-cells, more study is needed (17). On the contrary, studies have shown that there is no observed difference in mortality rates between patients with CM infected with COVID-19 and those without COVID-19 infection (18). Considering the potential overlap in symptoms between cryptococcal disease and COVID-19, rapid diagnosis of Cryptococcus through blood culture testing is crucial for prompt patient identification. Furthermore, ferroptosis has also been identified as playing a role in the pathogenesis of CM (19). In the field of CM, several studies have observed rapid iron accumulation and lipid peroxidation within the brain, both of which are hallmarks of ferroptosis, a type of programmed cell death characterized by iron dependence and lipid peroxidation (20). In recent years, numerous studies have confirmed the involvement of ferroptosis in various diseases, including infectious diseases like *Mycobacterium tuberculosis* infection and coronavirus disease-2019 (COVID-19) (6, 21). Furthermore, ferroptosis is considered immunogenic and pro-inflammatory as ferroptosis cells release damage-associated molecular pattern molecules (DAMPs) and alarmins, which both regulate immunity and pro-inflammatory activity (22). Therefore, we hypothesize that there may be a relationship between this unique cell death modality and CM. This suggests that alterations in the body’s immune function may increase the risk of infection.

The current treatment for CM often involves the combined use of antifungal medications, such as amphotericin B, flucytosine and fluconazole (23). The two patients respectively received treatment with amphotericin B and fluconazole, as well as amphotericin B and flucytosine, while concurrently using mannitol to reduce intracranial pressure, aiming to prevent complications, which is in line with the standard of care. The treatment had better efficacy for Case 2, whereas Case 1, due to multiple underlying conditions and delayed medical intervention, missed the treatment window and ultimately resulted in fatality. Past studies have involved successful cases of treating CM combined with COVID-19 infection using amphotericin B and flucytosine (12). but there have also been cases where treatment was not successful in saving the patients (24). We discuss these cases as presented in **Table 1**. These cases indicate that in immunocompromised patients, such as those with COVID-19, HIV infection, or organ transplants, caution should be exercised to avoid the use of corticosteroids or immunosuppressants, as this may increase the risk of developing fungal meningitis. Considering the widespread use of corticosteroids in severe COVID-19 infections, it is anticipated that there may be an increase in COVID-19 cases combined with fungal meningitis during the pandemic. When differentiating and diagnosing COVID-19-related brain disorders, CM should be considered as a primary concern, and prompt testing should be conducted accordingly.

**Table 1:** Clinical characteristics and treatment strategies for patients with concurrent COVID-19 and CM.

Study	Brief Summary	Infection type	Treatment Plan	Presence of other comorbid conditions	Clinical Outcome
Case 1 in this study	A 47-year-old male hospitalized for CM amidst the COVID-19 pandemic was subsequently diagnosed with COVID-19.	The coexistence of COVID-19 and CM.	The treatment course consisted of administering 200mg of Amphotericin B Lipid Complex once daily and combining it with 200mg of Fluconazole given twice daily. Due to disease progression, the subsequent treatment plan was changed to monotherapy with Voriconazole, and Remdesivir was added for antiviral therapy.	Hypertension; Diabetes Mellitus; Hyperthyroidism; Heart Disease	Death
Case 2 in this study	A 52-year-old male patient admitted to the hospital, during the course of management for CM, demonstrated strong indicators suggestive of a possible concurrent COVID-19 infection.	The patient presents with CM, strongly suspected to be complicated by a COVID-19 co-infection.	In the initial phase of treatment, the dosage of Amphotericin B is gradually increased from 30mg to a maintenance level of 200mg, while being co-administered with Flucytosine at a dose of 25mg per kg body weight, administered four times a day (QID). Moreover, supportive measures such as mannitol and albumin are utilized, and Molnupiravir is prescribed for antiviral treatment purposes.	Hypertension; Pancreatitis	Clinical improvement
Aghamali M et al., 2023 (25)	A 28-year-old male presenting with a seven-month progressive illness characterized by persistent fatigue, worsening fever, and headaches, recently complicated by the emergence of cough and chest pain, and having a history of interrupted treatment for a hematological disorder, has been definitively diagnosed with concurrent infections of COVID-19 and CM.	The coexistence of COVID-19 and CM.	Remdesivir as Antiviral Agent, Amphotericin B as Antifungal Agent, with Subsequent Use of Fluconazole and Tazocin.	HIV Infection, Pancytopenia,Diffuse Large B-Cell Lymphoma, Plasma Cell Dyscrasia.	Death
Abdessamad H W et al. 2023 (26)	A 76-year-old male developed fever and altered mental status following severe COVID-19 infection, which progressed to CM.	The coexistence of COVID-19 and CM.	Prednisone 40mg taken once daily (qd), reducing the dosage by 5mg every three days until reaching a maintenance dose of 5mg qd. Amphotericin B administered at a dose of 5mg/kg; Fluconazole given at a dose of 800mg qd.	Hypertension; Depression	Discharged after showing improvement.
Bongomin F et al. 2021 (27)	A 37-year-old female developed CM following severe COVID-19 infection.	The coexistence of COVID-19 and CM.	Dexamethasone 6mg qd; Enoxaparin 60mg qd; Paracetamol 1g three times daily(tid); Sulfamethoxazole-Trimethoprim combination 960mg qd; Fluconazole 1200mg qd; Piperacillin-Tazobactam 4.5g tid; Aspirin as a single loading dose of 300mg, followed by a maintenance dose of 75mg qd.	HIV Infection, Tuberculosis	Death
Alegre-González D et al. 2021 (28)	A 78-year-old male patient with severe COVID-19 underwent non-invasive ventilation and received high-dose corticosteroid therapy, after which *Cryptococcus neoformans* was isolated from his CSF.	The coexistence of COVID-19 and CM.	Dexamethasone 6mg qd for 3 days; Methylprednisolone 250mg qd for 3 days; Amphotericin B lipid complex at a dosage of 3mg/kg/24h and Flucytosine at a dosage of 25mg/kg/6h, which were later switched to Fluconazole 400 mg every 24 hours due to elevated creatinine levels.	Type 2 Diabetes Mellitus, Hypertension, Chronic Kidney Disease	Death
Thota D R et al. 2022 (15)	A 76-year-old female patient received immunosuppressive therapy during the course of treatment for severe COVID-19 pneumonia. Following her recovery phase, she subsequently developed symptoms of acute encephalopathy and multiple cerebral vascular obstructions, eventually being diagnosed with CM.	COVID-19 infection, subsequently complicated by CM.	Remdesivir; Tocilizumab at a dose of 4 mg/kg; methylprednisolone 40mg every 12 hours; Micafungin administered intravenously for 14 days; A 3-week course of intravenous Amphotericin B and Flucytosine Following negative fungal cultures from CSF, treatment was switched to oral fluconazole monotherapy via enteral route.	Hypertension, Osteoarthritis, Gastroesophageal Reflux Disease	In a persistent comatose state, transferred to a rehabilitation hospital.
Heller H M et al. 2020 (29)	A 26-year-old male with COVID-19 infection, subsequently tested positive for *Cryptococcus neoformans* in CSF.	The coexistence of COVID-19 and CM.	Intramuscular ketorolac was administered; Acetaminophen administered intravenously; empirical intravenous therapy with vancomycin, ceftriaxone, and acyclovir for broad-spectrum coverage. Mannitol was used to reduce intracranial pressure. Two weeks of treatment with amphotericin B and flucytosine, followed by consolidation and maintenance therapy using fluconazole.	HIV infection; status post appendectomy.	Discharged after showing improvement.
Ghanem H et al. 2021 (3)	A 73-year-old female, infected with COVID-19, developed new neurological symptoms and signs one week after receiving dexamethasone treatment, and *Cryptococcus neoformans* was detected in her CSF.	COVID-19 infection, subsequently complicated by CM.	Azithromycin and dexamethasone at a daily dose of 6mg; intravenous infusion of amphotericin B and flucytosine.	Not Applicable	Under ongoing treatment at a rehabilitation hospital.
Karnik K et al. 2022 (30)	A 57-year-old male with COVID-19 infection, tested positive for *Cryptococcus* in CSF during the hospitalization period.	COVID-19 infection, subsequently complicated by CM.	Dexamethasone 6mg qd, Remdesivir administered intravenously for 5 days; Liposomal Amphotericin B 400mg/day intravenously, and oral Flucytosine 2000 mg every other day.	Hypertension	Death
Liang D et al. 2024 (31)	A 57-year-old female, while undergoing immunosuppressive therapy for nephrotic syndrome, was found to have a COVID-19 infection accompanied by neurological symptoms, and *Cryptococcus* was detected in CSF.	COVID-19 infection, subsequently complicated by CM.	Dexamethasone; Favipiravir; Liposomal Amphotericin B (60mg qd) and Fluconazole (200mg bid).	Nephrotic Syndrome, Type 2 Diabetes Mellitus	Clinical improvement
Kim H et al. 2024 (24)	A 78-year-old female was hospitalized due to COVID-19 and subsequently developed CM following treatment.	COVID-19 infection, subsequently complicated by CM.	Administer Remdesivir intravenously, augmented with oral Baricitinib 4mg daily for a 2-week duration, concurrently utilizing intravenous Dexamethasone initially at a dose of 6mg, escalated to 12mg; followed by treatment with Amphotericin B and Flucytosine, and later transitioning to Fluconazole for consolidation antifungal therapy.	Hypertension, Hyperlipidemia, Diabetes Mellitus	Death
Hachey D M et al. 2023 (32)	A 69-year-old male with HIV infection was admitted to the hospital against the backdrop of the COVID-19 pandemic, and subsequent to his treatment, he developed CM.	CM with Suspected COVID-19.	Prednisone 20mg qd and Fluconazole 100mg qd; a single infusion of Liposomal Amphotericin B at a dose of 10 mg/kg (totaling 630mg), followed by Fluconazole 1200mg qd.	HIV infection; coronary artery disease, gastroparesis, and seasonal allergies	Clinical improvement

The two confirmed cases of CM underscore an elevated risk and severity of opportunistic infections in the context of the novel coronavirus. Even in subclinical conditions, the immune response remains active and may potentially trigger underlying or prior medical histories. This emphasizes the need for healthcare professionals to gain a deeper understanding of this condition and to offer comprehensive diagnosis and treatment. Regarding diagnosis, proficiency in laboratory testing methods is crucial, along with considering pathogenic infections, including less common opportunistic ones. Timely diagnosis and treatment play a crucial role in enhancing patient prognosis.

## Data availability statement

The original contributions presented in the study are included in the article/supplementary material. Further inquiries can be directed to the corresponding authors.

## Ethics statement

The studies involving human participants were reviewed and approved by the Ethics Committee of Hangzhou First People’s Hospital, Westlake University School of Medicine. The patients/participants provided written informed consent to participate in this study. Written informed consent for publication of this case report has been obtained from the participant/patient.

## Author contributions

YZ: Software, Resources, Writing – review & editing, Writing – original draft, Project administration, Methodology, Investigation, Funding acquisition, Formal analysis, Data curation, Conceptualization. BQ: Writing – original draft, Methodology, Conceptualization. JJiang: Software, Methodology, Writing – original draft. TC: Writing – original draft, Methodology, Formal analysis. LW: Supervision, Writing – review & editing. YY: Visualization, Writing – review & editing, Conceptualization. SR: Writing – original draft, Software, Formal analysis. YC: Writing – original draft, Project administration, Software. HF: Writing – original draft, Visualization, Resources, Conceptualization. JJ: Writing – review & editing, Methodology, Supervision, Conceptualization, Validation, Visualization. NY: Visualization, Supervision, Writing – review & editing, Writing – original draft, Methodology, Investigation, Conceptualization.
